# Cancer Therapy-Related Cardiac Dysfunction: A Review of Current Trends in Epidemiology, Diagnosis, and Treatment

**DOI:** 10.3390/biomedicines12122914

**Published:** 2024-12-21

**Authors:** Panagiotis Theofilis, Panayotis K. Vlachakis, Evangelos Oikonomou, Maria Drakopoulou, Paschalis Karakasis, Anastasios Apostolos, Konstantinos Pamporis, Konstantinos Tsioufis, Dimitris Tousoulis

**Affiliations:** 1 1st Department of Cardiology, Hippokration General Hospital, National and Kapodistrian University of Athens, 11527 Athens, Greece; panos.theofilis@hotmail.com (P.T.); vlachakispanag@gmail.com (P.K.V.); mdrakopoulou@med.uoa.gr (M.D.); anastasisapostolos@gmail.com (A.A.); konstantinospab@gmail.com (K.P.); ktsioufis@gmail.com (K.T.); 2 3rd Department of Cardiology, Thoracic Diseases General Hospital Sotiria, National and Kapodistrian University of Athens, 11527 Athens, Greece; boikono@gmail.com; 3 2nd Department of Cardiology, Hippokration General Hospital, Aristotle University of Thessaloniki, 54642 Thessaloniki, Greece; pakar15@hotmail.com

**Keywords:** cancer, cancer treatment-related cardiac dysfunction, global longitudinal strain, cardiac magnetic resonance, cardio-oncology

## Abstract

Cancer therapy-related cardiac dysfunction (CTRCD) has emerged as a significant concern with the rise of effective cancer treatments like anthracyclines and targeted therapies such as trastuzumab. While these therapies have improved cancer survival rates, their unintended cardiovascular side effects can lead to heart failure, cardiomyopathy, and arrhythmias. The pathophysiology of CTRCD involves oxidative stress, mitochondrial dysfunction, and calcium dysregulation, resulting in irreversible damage to cardiomyocytes. Inflammatory cytokines, disrupted growth factor signaling, and coronary atherosclerosis further contribute to this dysfunction. Advances in cardio-oncology have led to the early detection of CTRCD using cardiac biomarkers like troponins and imaging techniques such as echocardiography and cardiac magnetic resonance (CMR). These tools help identify asymptomatic patients at risk of cardiac events before the onset of clinical symptoms. Preventive strategies, including the use of cardioprotective agents like beta-blockers, angiotensin-converting enzyme inhibitors, mineralocorticoid receptor antagonists, and sodium-glucose cotransporter-2 inhibitors have shown promise in reducing the incidence of CTRCD. This review summarizes the mechanisms, detection methods, and emerging treatments for CTRCD, emphasizing the importance of interdisciplinary collaboration between oncologists and cardiologists to optimize care and improve both cancer and cardiovascular outcomes.

## 1. Introduction

The improvement in oncology care, particularly through the use of potent pharmacotherapies like anthracyclines and targeted agents such as trastuzumab, has dramatically enhanced cancer survival rates. However, these advances come with a significant risk: cancer therapy-related cardiac toxicity (CTRCD). As cancer treatments become more effective, their unintended cardiovascular side effects have become a critical issue. Indeed, while significant advances have been achieved in reducing cancer-related mortality, an increase in cardiovascular mortality is noted beyond the first years of cancer diagnosis [1].

Chemotherapeutic agents like anthracyclines generate reactive oxygen species (ROS), leading to oxidative stress and irreversible damage to cardiomyocytes [2]. Similarly, anti-vascular endothelial growth factor (VEGF) therapies used in treating solid tumors can cause hypertension and myocardial hypoperfusion, increasing the risk of heart failure (HF). With the emergence of powerful drugs, patients are often faced with the dual challenge of managing cancer while protecting cardiovascular health. Cardio-oncology, an interdisciplinary field that bridges oncology and cardiology, has become essential in managing this delicate balance. Early detection of cardiotoxicity through biomarkers like troponins and imaging modalities such as echocardiography and cardiac magnetic resonance (CMR) plays a pivotal role in identifying cardiovascular risks before irreversible damage occurs.

In response to the increasing prevalence of CTRCD, there is a growing focus on preventive and therapeutic strategies, including the use of cardioprotective agents like beta-blockers, angiotensin-converting enzyme (ACE) inhibitors, and angiotensin receptor blockers. These medications, when initiated early, can help mitigate the cardiovascular side effects of cancer therapy, allowing patients to continue potentially life-saving treatments. As the field of cardio-oncology continues to evolve, the goal is to optimize cancer treatment while minimizing its cardiovascular impact, ensuring both improved survival and quality of life for cancer patients. In this review, we summarize the existing evidence in the pathophysiology, epidemiology, and management of CTRCD. While multiple cancer therapies are associated with cardiac dysfunction, this review concerns mostly therapies with the most robust evidence for CTRCD, including anthracyclines, HER2-targeted therapies like trastuzumab, and anti-VEGF agents. These therapies are prioritized due to their widespread use and significant cardiovascular impact in clinical practice.

## 2. Definition

Cardiovascular toxicities related to cancer therapies encompass a broad spectrum of conditions that affect the heart and vascular system. These manifestations necessitate cross-disciplinary communication to effectively describe cardiovascular events and integrate these standards into routine clinical practice and research. The recently published 2022 ESC Guidelines for the management of cardio-oncology, endorsed by the Scientific Council of the International Cardio-Oncology Society (IC-OS), provide comprehensive definitions of these toxicities [3].

More specifically, “Cancer Therapy-Related Cardiac Dysfunction” (CTRCD) is defined as the adverse impact on cardiac structure and function in cancer patients, presenting as either asymptomatic cardiac dysfunction or symptomatic HF related to the therapy received. Confirmation of CTRCD includes any of the following: a reduction in left ventricular ejection fraction (LVEF), symptoms of congestive heart failure, signs associated with heart failure (such as S3 gallop or tachycardia), or a reduction in LVEF from baseline by 5–10%. Symptomatic CTRCD is characterized by a HF syndrome with typical symptoms and signs of volume overload or inadequate perfusion caused by structural and functional abnormalities of the heart consistent with AHA/ACC Stage C/D HF. In contrast, asymptomatic CTRCD is more common during cancer therapy and is often identified through changes in LVEF on screening echocardiograms during treatment or as an incidental finding during survivorship surveillance [4].

The guidelines also define various manifestations of cancer therapy-related cardiac toxicity. Cardiomyopathy and HF are significant conditions that can arise, with myocarditis often resulting from direct toxicity or immune-mediated events in cancer patients. Vascular toxicity, characterized by arterial occlusion due to blood clots, can lead to luminal obstruction and reduced blood flow through altered vascular reactivity, thrombosis, atherosclerosis, or vasculitis, and can be either clinically silent or symptomatic. Hypertension in cancer patients may be temporary and treatment-induced, with a more abrupt onset that can lead to end-organ damage and other complications. Uncontrolled hypertension may necessitate the cessation of cancer treatment, significantly impacting the patient’s oncologic care. Finally, several cancer therapies have been recognized to cause QTc prolongation, including arsenic trioxide, HDAC inhibitors, tyrosine kinase inhibitors (e.g., vandetanib, vemurafenib, ceritinib, gilteritinib, trametinib, and those targeting BCR-Abl and the VEGF signaling pathway), and Cyclin-dependent kinase (CDK) 4–6 inhibitors [4].

Understanding these definitions and mechanisms is crucial, as “speaking the same language” among clinicians could improve the management of cardiovascular health in cancer patients and ensure optimal therapeutic outcomes.

## 3. Epidemiology

The epidemiology of cardiotoxicity varies significantly depending on the population studied and the specific agents involved. In oncology, the incidence of cardiotoxicity is influenced by the type of chemotherapeutic agent, cumulative dose, combination therapies, and individual patient risk factors such as age, pre-existing cardiovascular disease, and genetic predispositions.

Specifically, the prevalence of late symptomatic anthracycline-induced cardiotoxicity varies widely, influenced by patient age, anthracycline dose, cancer type, cardiovascular risk factors, pre-existing heart disease, and follow-up duration [5]. In adults, symptomatic HF typically appears within two to three years after anthracycline treatment [6]. A study of 135 patients with non-Hodgkin lymphoma showed that 20% experienced significant cardiac events within a year of anthracycline therapy [7]. Asymptomatic LV dysfunction is more common than symptomatic disease, with rates ranging from 7% (LVEF) to 45% (cardiac strain) [8]. A trial on statin therapy in doxorubicin-treated patients showed a decrease in LVEF from 63% to 57% over 24 months [9]. In an echocardiographic study of 1853 adult childhood cancer survivors, 7% had asymptomatic LVEF reduction below 50% [10]. A CMR imaging study reported a 14% prevalence of reduced LVEF in 114 adult childhood cancer survivors [11]. Another CMR study found 26% of adults treated with low to moderate anthracycline doses had an asymptomatic LVEF reduction below 50% at six months [12]. A Kaiser study linked anthracycline therapy for breast cancer to a 1.84-fold increased risk of cardiomyopathy/HF and a 2.91-fold increased risk of cardiovascular mortality [13]. In children, cardiotoxicity is often detected long after exposure, particularly in childhood cancer survivors, who have a nearly 6-fold higher risk of HF compared to siblings, mainly due to anthracycline exposure [10].

The incidence of trastuzumab-related cardiotoxicity varies based on patient factors such as previous chemotherapy, pre-existing heart disease, and age. While the risk of HF or cardiomyopathy with trastuzumab is low, it can be mitigated by limiting cumulative anthracycline doses to below 300 mg/m^2^. Nevertheless, close cardiac monitoring is necessary [14]. In large randomized trials of adjuvant trastuzumab for HER2-positive breast cancer, which included stringent cardiac monitoring and limited anthracycline doses, a modest incidence of cardiotoxicity was observed [15,16,17]. A 2012 meta-analysis of these trials, involving 11,991 women, found that trastuzumab-treated patients had an increased risk of severe HF (2.5% vs. 0.4%; RR 5.11) and a reduction in LVEF (RR 1.83). The cardiotoxicity profile was similar regardless of whether chemotherapy and trastuzumab were administered concurrently or sequentially. Shorter treatment periods did not significantly increase HF risk [18]. A subsequent study of around 400 patients treated with paclitaxel and trastuzumab for node-negative disease reported even lower cardiotoxicity rates, with only 0.5% developing grade 3 left ventricular systolic dysfunction and 3% experiencing asymptomatic LVEF decline. These findings compare favorably with older trials involving higher doses of concurrent doxorubicin [19,20].

The incidence of fluorouracil (FU)-related cardiotoxicity ranges from 1% to 19%, with most reports indicating a risk of 8% or less [21]. This variability is likely due to differences in defining cardiotoxicity, FU administration methods, underlying coronary artery disease (CAD), concurrent radiation therapy or anthracyclines, and monitoring intensity. The highest rates are seen in closely monitored patients. For instance, a study of 102 patients receiving FU, monitored with ECG, echocardiography, and radionuclide ventriculography, found 19% experienced reversible angina pectoris within 24 h of starting FU, often with accompanying ECG changes [22]. Asymptomatic ECG changes, arrhythmias, and elevated NT-proBNP levels during FU chemotherapy suggest possible subclinical cardiotoxicity, though the clinical significance is uncertain [22,23,24].

Tyrosine kinase inhibitors (TKIs) are associated with significant cardiovascular toxicity, affecting both the heart and vascular system [25]. Adverse effects are observed in single- and multi-targeted TKIs, particularly through mechanisms such as VEGF inhibition, which impairs angiogenesis and myocardial perfusion and leads to microvascular dysfunction [25]. Myocardial dysfunction and HF are common, with a meta-analysis of clinical studies in 10,670 patients reporting an incidence of asymptomatic LV dysfunction at 2.4%, while VEGF inhibitors like sunitinb and sorafenib are linked to progressive HF [26]. Specifically, VEGF inhibitors can reduce capillary rarefaction (myocardial capillary network) and impaired myocardial contractility [27]. Anti-HER2 TKIs, such as lapatinib, show higher cardiotoxicity rates in metastatic settings, with HF reported in 3% of patients with breast cancer [28]. HTN is another prevalent side effect, which is seen in 30–80% of patients on VEGFIs and in up to 78.3% of those treated with ibrutinib [25,29]. QTc prolongation occurs in 0.1% of VEGF-inhibitor cases but is higher with third-generation EGFR TKIs such as osimertinib [30,31]. Finaly, thromboembolic events are associated with VEGF inhibitors, with arterial thrombosis reported in 1.7% and 1.4% of patients on sorafenib and sunitinib, respectively [25,32].

Immune checkpoint inhibitors (ICIs) are associated with rare but significant cardiotoxicity, primarily presenting as myocarditis. A retrospective study across eight clinical centers found a myocarditis prevalence of 1.14% [33]. In a phase II trial with pembrolizumab for thymic epithelial tumors, 34% of patients experienced grade 3 or higher immune-related adverse events, including 9% with myocarditis and 6% with myasthenia gravis [34]. Combination ICI therapy increases myocarditis risk to 1.33%, compared to 0.31% for monotherapy [35]. Myocarditis typically manifests within the first three months, with most cases occurring within six weeks of treatment, and combination therapy-related myocarditis shows a higher mortality rate (67%) compared to monotherapy (36%) [36]. Other CV complications include a 1% incidence of myocardial infarction in lung cancer patients treated with ICIs and tachyarrhythmias, bradyarrhythmias, and prolonged QTc, indicating an elevated risk of ventricular arrhythmias [37,38].

## 4. Pathophysiology

**Figure 1:**
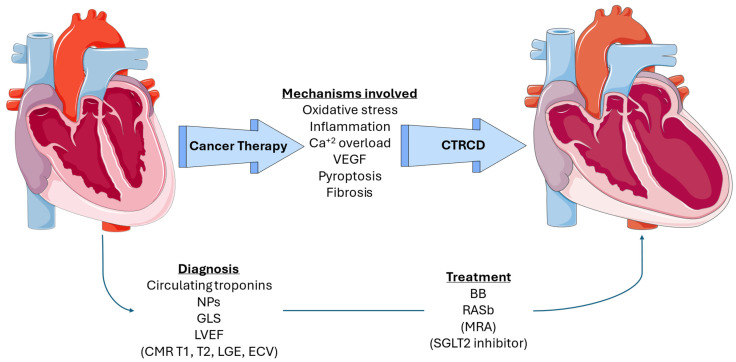
Overview of pathophysiologic mechanisms, diagnosis, and treatment of CTRCD. Content in parenthesis represents potential approaches that have not been incorporated into guidelines. VEGF: vascular endothelial growth factor, NP: natriuretic peptide, GLS: global longitudinal strain, LVEF: left ventricular ejection fraction, CMR: cardiac magnetic resonance, LGE: late gadolinium enhancement, ECV: extracellular volume, BB: beta blocker, RASb: renin-angiotensin system blocker, MRA: mineralocorticoid receptor antagonist, and SGLT2: sodium-glucose cotransporter-2.

The pathophysiology of cardiotoxicity involves complex mechanisms that result in myocardial damage (Table 1, Figure 1). For chemotherapeutic agents like anthracyclines, the primary mechanism is believed to be the generation of ROS leading to oxidative stress, mitochondrial dysfunction, and subsequent cardiomyocyte apoptosis [39]. In cardiomyocytes, which have abundant mitochondria, ROS levels increase during chemotherapy, predominantly producing superoxide anions [40]. Due to their short half-life, ROS primarily affect the mitochondria that generate them [41]. Additionally, the heart’s crucial antioxidant systems, which neutralize free radicals, are diminished [42]. This includes reduced complex I activity and decreased levels of cardiolipin, a phospholipid essential for the function of anion carriers and electron transport complexes, as well as for enhancing cytochrome oxidase activity [43]. Cardiolipin, an unsaturated fatty acid vital for electron transport and cytochrome oxidase activity, is prone to peroxidation, leading to decreased cytochrome c oxidase activity and increased oxidative stress [44]. Oxidative stress in the cardiac muscle can lead to altered gene expression, calcium overload, cellular hypertrophy, ventricular remodeling, and cell death via apoptosis. These pathological changes can result in cardiomyopathy and HF [40].

Moreover, chronic inflammation and cytokine storming, often perpetuated by malignancy and chemotherapy, could have deleterious effects on the cardiovascular system. Inflammatory cytokines such as Interleukin (IL)-1β and IL-6 alter cardiac ion channel function, increasing the risk of arrhythmias and sudden cardiac death [45]. For instance, increased inflammatory markers inhibit human ether-a-go-go (hERG)-related channel, reducing the rapid delayed rectifier current (IKr), prolonging the QT interval and action potential duration, and heightening the risk of ventricular arrhythmias like Torsades de Pointes [46,47]. Key ion channels, including SERCA2 and Cav1.2 (L-type Ca^2+^ channels), are affected by elevated levels of IL-6 and IL-1 [55]. Chronic exposure to IL-6 has been shown to decrease SERCA2 activity, impairing Ca^2+^ reuptake and leading to prolonged Ca^2+^ exposure, sustained excitation, and an increased risk of arrhythmias [56,57]. Additionally, IL-6 plays a complex role in myocardial contractility [58]. While acute IL-6 exposure can help preserve myocardial function, chronic exposure can lead to pathological changes, including immune cell infiltration and increased connective tissue production. Continuous IL-6 and IL-6 receptor expression can cause persistent gp130 signaling, promoting left ventricular hypertrophy and cardiomyopathy [59]. STAT-3, a downstream target of IL-6, is implicated in cardiac hypertrophy, while tumor necrosis factor (TNF)-α has been observed to reduce ejection fraction and induce HF in animal studies [58,60]. IL-1β and IL-18 also promote apoptosis by increasing caspase and gasdermin activity, contributing to myocardial pyroptosis, a form of necrosis associated with excessive cytokine release during ischemia–reperfusion injury [53].

Calcium regulation within the myocardium is crucial for muscle contractility and conduction in the atrioventricular and sinoatrial nodes. Excess calcium can lead to increased firing of these nodes, and any pathological alterations in calcium handling can result in cardiotoxicity, which is a known mechanism of chemotherapy-induced cardiotoxicity. Certain chemotherapeutic drugs significantly affect calcium homeostasis in cardiac tissue. Studies suggest that these drugs increase ROS levels, which subsequently cause calcium overload [48]. For instance, doxorubicin has been shown to increase diastolic calcium overload in rat cardiac myocytes by promoting calcium leakage from the sarcoplasmic reticulum through modulation of Ca/calmodulin-dependent protein kinase II (CaMKII) activity [49]. Lowering ROS levels, reducing Na^+^/Ca^2+^ exchanger expression, and preventing CaMKII hyperphosphorylation can mitigate anthracycline-induced myocardial fibrosis [61]. Excess calcium can lead to fatal atrial and ventricular arrhythmias, with common ECG findings of early and delayed afterdepolarizations. Calcium overload, often due to ryanodine receptor 2 (RYR2) dysfunction, can create ectopic foci and reentry circuits, disrupting cardiac conduction [50].

Growth factors are essential for cell proliferation and integrity. VEGF supports angiogenesis, myocardial regeneration, and stress response by upregulating nitric oxide, which inhibits platelet adhesion and acts as a vasodilator [51]. However, VEGF also promotes tumor growth and metastasis, leading to the use of anti-VEGF therapies in cancers like breast, lung, and colorectal cancer. Anti-VEGF drugs can cause significant cardiotoxicity, with up to 70% of patients developing arterial hypertension [52]. This is possibly due to increased endothelin-1 levels and higher systolic blood pressure, which can lead to cardiac remodeling, LV hypertrophy, diastolic dysfunction, and HF. Similarly, inhibiting platelet-derived growth factors (PDGFR) with drugs like sunitinib can cause hypertrophy and fibrosis [54]. Epidermal growth factor receptor inhibitors (EGFRis) also contribute to cardiotoxicity, with some patients experiencing severe HF when treated with herceptin and anthracyclines. Despite their benefits, the cardiotoxicity of anti-growth signaling therapies limits their use [62].

Finally, several chemotherapeutic drugs are implicated in atherosclerosis of the coronary arteries. Antimetabolites, antimicrotubule agents, and tyrosine kinase inhibitors commonly cause coronary artery disease due to alterations in signaling pathways and subsequent vasoconstrictive effects. VEGF inhibitors can lead to coronary artery occlusion and angina by reducing NO synthase activity via the Akt/PKB pathway, resulting in abnormal vascular tone and coagulation, especially during oxidative stress [27]. Combined with the increased coagulative tendency and the effects of radiotherapy in malignancy, these factors may significantly impact cardiovascular health and quality of life in cancer patients.

## 5. The Role of Biomarkers and Imaging in CTRCD

Biomarkers of cardiac dysfunction are crucial in the detection and subsequent management of CTRCD (Table 2). Early studies have shown that a rise in TnT following anticancer therapy (chemotherapy or radiotherapy) was associated with the incidence of CTRCD and is usually dose dependent [63,64]. According to a meta-analysis of eight studies (1294 patients receiving anticancer therapy), an increase in TnT at 3–6 months after the initiation of treatment was an strong predictor of CTRCD (area under receiver operating characteristics curve 0.90) [65]. It is therefore unsurprising that a rise in troponin irrespective of symptoms could indicate the presence of mild, asymptomatic CTRCD, according to the guidelines, and should be ordered in all patients prior to the initiation of anticancer therapy, even in those in the lowest risk categories.

Regarding imaging modalities, it is clear that echocardiography remains a first-line imaging modality in assessing CTRCD, either through the use of GLS or LVEF, as proposed by the existing guidelines, since it is a cost-effective and reproducible method. Recently, CMR has flourished in this field due to its ability in myocardial tissue characterization and detection of myocardial inflammation and fibrosis.

Changes in epicardial adipose tissue could be an early indicator of incident adverse cardiac remodeling, as shown in a previous study [71]. Chest computed tomography (CT) in patients receiving cardiotoxic chemotherapy has shown an expansion in the volume of epicardial adipose tissue [66]. Therefore, future studies will determine whether this imaging modality can detect patients at risk of CTRCD at an earlier stage and prompt cardioprotective treatment initiation. CT-derived myocardial extracellular volume could also present an appealing marker which requires further validation, since a small-scale study in 82 breast cancer patients found it to be an informative marker [67].

Cardiac magnetic resonance (CMR) has recently gained ground regarding the early recognition of CTRCD. A study evaluated the early detection of anthracycline-induced cardiotoxicity in breast cancer survivors using T2 CMR [68]. It revealed that myocardial T2, which indicates inflammation, increases after anthracycline therapy, preceding fibrosis and a decline in LVEF. Though LVEF remained stable initially, 35% of patients showed a significant drop in LVEF at follow-up, suggesting T2 as an early marker of cardiotoxicity. In another study in women undergoing breast cancer therapy with anthracyclines and trastuzumab, CMR markers of myocardial hyperemia and edema (relative myocardial enhancement, native T1, extracellular volume) increased after anthracycline chemotherapy or 3 months after trastuzumab treatment, indicating an inflammatory process during this period [69]. Moreover, they were associated with increased LV volumes and BNP levels but the changes were transient and not associated with the incidence of CTRCD. For cancer patients treated with ibrutinib for a median of 14 months, CMR late gadolinium enhancement (LGE) was present in 13.3% and indices of subclinical myocardial inflammation and fibrosis were found to be elevated in 63% and 28.6% of patients, respectively [70]. Interestingly, LGE (HR: 4.9; *p* = 0.04) and native-T1 (HR: 3.3; *p* = 0.05) were predictive of incident adverse cardiovascular events (atrial fibrillation, heart failure, symptomatic ventricular arrhythmias, and sudden death). We should note that according to a study in 125 women with HER2+ early-stage breast cancer receiving sequential anthracycline/trastuzumab, echocardiography-derived GLS was the most optimal prognostic marker even when compared to feature-tracking-derived GLS and global circumferential strain from CMR [72].

Given the inconclusive evidence to date, there is a need for further observational and randomized studies to determine the true potential of CMR in this setting.

## 6. Prevention and Treatment of CTRCD

As it has become evident that patients with cancer are at a heightened risk of developing cardiovascular disease. Given the existing knowledge in the pathophysiology and the presence of biomarkers for diagnosis and prognosis of CTRCD, research in the field is now focused on ways of preventing and treating CTRCD. In the field of prevention, several studies have been conducted. In patients diagnosed with cancer who had an indication to receive anthracycline chemotherapy, enalapril was tried as a preventive measure at the first chemotherapy cycle, while a control group received the medication at the time of troponin elevation during chemotherapy [73]. According to the results of this trial, the incidence of a troponin rise was similar across the two groups. Another study utilized the beta-blocker carvedilol in patients with HER2-negative breast cancer receiving anthracycline-containing treatment [74]. The authors found no differences in LVEF or troponin I levels between the carvedilol and placebo groups. The PRADA study compared multiple CTRCD prevention regimens in patients with early breast cancer [75]. At a median follow-up of 23 months, no differences in LVEF were noted in the studied groups (candesartan, metoprolol, placebo). Similarly, cardiac biomarkers (TnI, TnT, NTproBNP) were not significantly different with regards to treatment. Candesartan further failed to prevent cardiac events at a 2-year follow-up in 210 women with HER+ breast cancer who received anthracycline and trastuzumab as part of their chemotherapy [76]. It also did not affect cardiac biomarkers or the changes in LVEF compared to placebo. Among the promising agents in the prevention of CTRCD are mineralocorticoid receptor antagonists. Akpek et al., by utilizing a sample of 83 patients randomized to spironolactone or placebo, showed a significantly slower decline in LVEF with spironolactone, accompanied by steeper TnI rise and unaltered NTproBNP levels compared to placebo [77].

Treating CTRCD is another challenging task, often mandating the use of medications used for patients with HF. In the presence of mild, asymptomatic CTRCD that is indicated by abnormalities in GLS, TnI, or NTproBNP, the use of renin-angiotensin system blockers with or without beta-blockers is indicated by the existing European Society of Cardiology Cardio-oncology guidelines (Table 3) [3]. At least moderate and asymptomatic, or symptomatic CTRCD mandates the use of HF medications by following the related treatment algorithms [3]. For SGLT2 inhibitors in particular, Gongora et al. showed that the incidence of cardiac events (heart failure incidence-hospitalization, cardiotoxicity, clinically significant arrhythmias) was lower in diabetic patients receiving SGLT2 inhibitors compared to the control group [78]. A recent, larger-scale study compared the use of guideline-directed medical therapy with or without an SGLT2 inhibitor in patients with type 2 DM and CTRCD [79]. In a population of 6988 patients (654 used an SGLT2 inhibitor), the authors found a significantly lower incidence of all-cause mortality (OR: 0.296 [95% CI: 0.22–0.40]; *p* = 0.001) and HF decompensation (OR: 0.483 [95% CI: 0.36–0.65]; *p* < 0.001). Secondary endpoints were also favoring the use of SGLT2 inhibitors, namely the incidence of atrial fibrillation/flutter (OR: 0.397 [95% CI: 0.213–0.737]; *p* = 0.003), acute kidney injury (OR: 0.486 [95% CI: 0.382–0.619]; *p* < 0.001), and the need for renal replacement therapy (OR: 0.398 [95% CI: 0.189–0.839]; *p* = 0.012).

## 7. Future Directions-Gaps in Evidence

Despite significant progress in the field of cardio-oncology, several critical gaps in knowledge and clinical practice remain. Early detection of CTRCD is a pressing challenge. Current biomarkers, such as troponins and NT-proBNP, are reliable but often detect cardiac injury after irreversible dysfunction has occurred. Novel biomarkers, including galectin-3 and soluble ST2 (sST2), hold promise in identifying early myocardial stress or fibrosis but require further validation in large-scale clinical trials. Additionally, imaging modalities such as CMR and CT-derived extracellular volume measurements offer exciting potential for earlier detection of subclinical cardiac injury. However, studies are needed to define the optimal timing, cost-effectiveness, and real-world applicability of these advanced imaging tools.

Therapeutic innovations in CTRCD prevention also remain underexplored. While medications like spironolactone and SGLT2 inhibitors have shown potential in mitigating cardiac dysfunction, most evidence stems from small-scale studies. Multicenter, randomized controlled trials are urgently needed to confirm the effectiveness of these agents in broader populations. Similarly, while traditional cardioprotective agents such as beta-blockers and angiotensin-converting enzyme inhibitors are widely used, their role in specific high-risk subgroups or in combination with newer therapies has yet to be fully delineated. Investigating the interaction between cancer therapies and cardioprotective drugs, particularly in complex cases involving multiple comorbidities, should be prioritized to refine treatment protocols.

Personalized risk prediction and management represent another significant area for advancement. Current approaches to CTRCD prevention often rely on population-based strategies, which may overlook the heterogeneity of patient responses to cancer therapies. Developing precision medicine tools that integrate clinical, genetic, and treatment-specific factors can improve the prediction of CTRCD risk. Leveraging artificial intelligence and machine learning algorithms to analyze large datasets from clinical trials and registries could provide actionable insights for individualized management strategies. Such models could also help identify high-risk patients who would benefit most from intensive cardiac monitoring or early cardioprotective interventions.

Finally, fostering interdisciplinary collaboration and expanding access to cardio-oncology resources are essential to addressing gaps in care. Establishing international cardio-oncology registries could provide valuable real-world data on CTRCD incidence, treatment outcomes, and best practices. Additionally, efforts to adapt guidelines, such as those from the European Society of Cardiology (ESC), to diverse healthcare settings—including resource-limited environments—are critical for ensuring equitable care. Addressing implementation barriers, such as training for healthcare professionals and the financial burden of advanced diagnostic tools, will be crucial in optimizing outcomes for cancer patients globally. By addressing these challenges, future research and clinical efforts can ensure that advancements in cancer care are not achieved at the expense of cardiovascular health.

## 8. Conclusions

Cancer therapy-induced cardiotoxicity presents a growing concern as cancer survival rates improve. This adverse effect, particularly common with anthracyclines and targeted therapies like trastuzumab, underscores the need for a balance between effective cancer treatment and cardiovascular safety. Early detection using cardiac biomarkers and advanced imaging techniques, combined with preventive strategies like the use of beta-blockers or ACE inhibitors, can mitigate long-term cardiac damage. The evolution of cardio-oncology highlights the importance of an interdisciplinary approach, where collaboration between oncologists and cardiologists is key to reducing cardiovascular risks. As research advances, the development of targeted cardioprotective therapies offers promising avenues to safeguard patients’ heart health without compromising cancer treatment efficacy. Ensuring continuous monitoring and personalized interventions will be crucial to improving both oncological and cardiovascular outcomes for cancer survivors.

## Figures and Tables

**Table 1:** Mechanisms of cancer therapy-related cardiac dysfunction (CTRCD).

Mechanism	Drug Class	Observed Symptoms	Key Processes	References
Oxidative Stress	Anthracyclines	Cardiomyopathy, heart failure	ROS generation, mitochondrial dysfunction, cardiomyocyte apoptosis	[39,40,43,44]
Inflammation	Immune checkpoint inhibitors	Myocarditis, arrhythmias	Cytokine storm, IL-1β and IL-6 mediated ion channel alterations, QT prolongation	[45,46,47]
Calcium Overload	Anthracyclines, TKIs	Ventricular arrhythmias, myocardial injury	Disruption of calcium regulation, increased diastolic calcium levels, RYR2 dysfunction	[48,49,50]
VEGF	Anti-VEGF therapies	Hypertension, myocardial ischemia	Endothelial dysfunction, impaired angiogenesis, microvascular dysfunction	[27,51,52]
Pyroptosis	Immune checkpoint inhibitors	Myocardial necrosis, inflammation	Caspase and gasdermin activation, increased cytokine release during ischemia– reperfusion injury	[53]
Fibrosis	HER2-targeted therapies	Myocardial stiffening, heart failure	Increased fibroblast activation, extracellular matrix remodeling, TGF-β pathway activation	[12,54]

ROS: reactive oxygen species, IL: interleukin, TKI: tyrosine kinase inhibitor, VEGF: vascular endothelial growth factor, RYR2: ryanodine receptor 2.

**Table 2:** Circulating and novel imaging biomarkers in CTRCD.

Biomarker	Anticancer Therapy	Study Population	Outcomes	Ref
Hs-cTnT	Platinum and taxane-doublet chemotherapy with radiation of at least 60 Gy	190 patients with NSCLC	Hs-cTnT increased from 4th week Greater increase in left-sided tumors Δhs-cTnT correlated with mean heart dose Risk of cardiac adverse events higher if baseline hs-cTnT >10 ng/L (HR 4.06) or Δ-hs-cTnT >5 ng/L (HR 3.57)	[63]
Hs-cTnT	Not specified	930 patients attending the cardio-oncology outpatient clinic	hs-cTnT above the median (≥7 ng/L) was an independent marker of all-cause mortality (OR 2.21)	[64]
CT-derived EAT volume index	Adjuvant AC ± Trastuzumab	41 breast cancer patients	The EAT volume index was significantly higher in both groups at follow-up compared to baseline	[66]
CT-derived ECV	Neoadjuvant chemotherapy	102 breast cancer patients	Development of CTRCD was associated with an increase in mean ECV after approximately 12 months, which had subsided at 5 years	[67]
Myocardial T2	Anthracycline	29 breast cancer patients	Gradual increase of T2 values during chemotherapy	[68]
T1, T2, and ECV	Anthracycline + Trastuzumab	136 breast cancer patients	Changes in ventricular volumes were associated with a change in T1, T2, and/or ECV No significant associations were seen between any of the CMR tissue biomarkers and LVEF, GLS, or LVMi Larger increase in T1, T2, and ECV was associated with a larger increase in BNP	[69]
CMR-LGE	Ibrutinib	49 cancer patients	Native T1 and presence of LGE were higher in treated patients and were associated with the development of MACE (LGE HR: 4.9, T1 HR: 3.3)	[70]

**Table 3:** Summary of the ESC guideline recommendations for the management of CTRCD associated with anthracyclines and anti-HER2 agents [3].

Symptomatic	CTRCD Severity	Recommendation (Class/Level)
AC		
Yes	Severe	HF therapy (I/B) Discontinue AC (I/C)
Yes	Moderate	HF therapy (I/B) Interrupt AC (I/C)
Yes	Mild	HF therapy (I/B) MDT for interruption vs. continuation (I/C)
No	Severe/Moderate	HF therapy (I/B) Interrupt AC (I/C)
No	Mild	Continue AC (I/C) ACEi/ARB and/or BB if GLS decreases/TnI increases (IIa/B) or NP increases (IIb/C)
Anti-HER2		
Yes	Severe/Moderate	HF therapy (I/B) Interrupt anti-HER2
Yes	Mild	HF therapy (I/B) MDT for interruption vs. continuation (I/C)
No	Severe	HF therapy (I/B) Interrupt anti-HER2
No	Moderate	Continue anti-HER2 (IIa/B) HF therapy (I/B)
No	Mild	Continue anti-HER2 (I/C) ACEi/ARB and/or BB if GLS decreases or TnI/NP increase (IIa/B)

AC: anthracycline, HF: heart failure, MDT: multidisciplinary team, ACEi: angiotensin-converting enzyme inhibitor, ARB: angiotensin receptor blocker, BB: beta blocker, GLS: global longitudinal strain, TnI: troponin I, and NP: natriuretic peptide.

## References

[1] Stoltzfus K.C., Zhang Y., Sturgeon K., Sinoway L.I., Trifiletti D.M., Chinchilli V.M., Zaorsky N.G. (2020). Fatal heart disease among cancer patients. Nat. Commun..

[2] Suter T.M., Ewer M.S. (2013). Cancer drugs and the heart: Importance and management. Eur. Heart J..

[3] Lyon A.R., Lopez-Fernandez T., Couch L.S., Asteggiano R., Aznar M.C., Bergler-Klein J., Boriani G., Cardinale D., Cordoba R., Cosyns B. (2022). 2022 ESC Guidelines on cardio-oncology developed in collaboration with the European Hematology Association (EHA), the European Society for Therapeutic Radiology and Oncology (ESTRO) and the International Cardio-Oncology Society (IC-OS). Eur. Heart J..

[4] Herrmann J., Lenihan D., Armenian S., Barac A., Blaes A., Cardinale D., Carver J., Dent S., Ky B., Lyon A.R. (2022). Defining cardiovascular toxicities of cancer therapies: An International Cardio-Oncology Society (IC-OS) consensus statement. Eur. Heart J..

[5] Wang L., Tan T.C., Halpern E.F., Neilan T.G., Francis S.A., Picard M.H., Fei H., Hochberg E.P., Abramson J.S., Weyman A.E. (2015). Major Cardiac Events and the Value of Echocardiographic Evaluation in Patients Receiving Anthracycline-Based Chemotherapy. Am. J. Cardiol..

[6] Bostany G., Chen Y., Francisco L., Dai C., Meng Q., Sparks J., Sessions M., Nabell L., Stringer-Reasor E., Khoury K. (2024). Cardiac Dysfunction Among Breast Cancer Survivors: Role of Cardiotoxic Therapy and Cardiovascular Risk Factors. J. Clin. Oncol..

[7] Limat S., Demesmay K., Voillat L., Bernard Y., Deconinck E., Brion A., Sabbah A., Woronoff-Lemsi M.C., Cahn J.Y. (2003). Early cardiotoxicity of the CHOP regimen in aggressive non-Hodgkin’s lymphoma. Ann. Oncol..

[8] Sawaya H., Sebag I.A., Plana J.C., Januzzi J.L., Ky B., Tan T.C., Cohen V., Banchs J., Carver J.R., Wiegers S.E. (2012). Assessment of echocardiography and biomarkers for the extended prediction of cardiotoxicity in patients treated with anthracyclines, taxanes, and trastuzumab. Circ. Cardiovasc. Imaging.

[9] Hundley W.G., D’Agostino R., Crotts T., Craver K., Hackney M.H., Jordan J.H., Ky B., Wagner L.I., Herrington D.M., Yeboah J. (2022). Statins and Left Ventricular Ejection Fraction Following Doxorubicin Treatment. NEJM Evid..

[10] Mulrooney D.A., Yeazel M.W., Kawashima T., Mertens A.C., Mitby P., Stovall M., Donaldson S.S., Green D.M., Sklar C.A., Robison L.L. (2009). Cardiac outcomes in a cohort of adult survivors of childhood and adolescent cancer: Retrospective analysis of the Childhood Cancer Survivor Study cohort. BMJ.

[11] Armstrong G.T., Plana J.C., Zhang N., Srivastava D., Green D.M., Ness K.K., Daniel Donovan F., Metzger M.L., Arevalo A., Durand J.B. (2012). Screening adult survivors of childhood cancer for cardiomyopathy: Comparison of echocardiography and cardiac magnetic resonance imaging. J. Clin. Oncol..

[12] Drafts B.C., Twomley K.M., D’Agostino R., Lawrence J., Avis N., Ellis L.R., Thohan V., Jordan J., Melin S.A., Torti F.M. (2013). Low to moderate dose anthracycline-based chemotherapy is associated with early noninvasive imaging evidence of subclinical cardiovascular disease. JACC Cardiovasc. Imaging.

[13] Greenlee H., Iribarren C., Rana J.S., Cheng R., Nguyen-Huynh M., Rillamas-Sun E., Shi Z., Laurent C.A., Lee V.S., Roh J.M. (2022). Risk of Cardiovascular Disease in Women With and Without Breast Cancer: The Pathways Heart Study. J. Clin. Oncol..

[14] Henry M.L., Niu J., Zhang N., Giordano S.H., Chavez-MacGregor M. (2018). Cardiotoxicity and Cardiac Monitoring Among Chemotherapy-Treated Breast Cancer Patients. JACC Cardiovasc. Imaging.

[15] Bria E., Cuppone F., Fornier M., Nistico C., Carlini P., Milella M., Sperduti I., Terzoli E., Cognetti F., Giannarelli D. (2008). Cardiotoxicity and incidence of brain metastases after adjuvant trastuzumab for early breast cancer: The dark side of the moon? A meta-analysis of the randomized trials. Breast Cancer Res. Treat..

[16] Slamon D., Eiermann W., Robert N., Pienkowski T., Martin M., Press M., Mackey J., Glaspy J., Chan A., Pawlicki M. (2011). Adjuvant trastuzumab in HER2-positive breast cancer. N. Engl. J. Med..

[17] Gianni L., Eiermann W., Semiglazov V., Manikhas A., Lluch A., Tjulandin S., Zambetti M., Vazquez F., Byakhow M., Lichinitser M. (2010). Neoadjuvant chemotherapy with trastuzumab followed by adjuvant trastuzumab versus neoadjuvant chemotherapy alone, in patients with HER2-positive locally advanced breast cancer (the NOAH trial): A randomised controlled superiority trial with a parallel HER2-negative cohort. Lancet.

[18] Moja L., Tagliabue L., Balduzzi S., Parmelli E., Pistotti V., Guarneri V., D’Amico R. (2012). Trastuzumab containing regimens for early breast cancer. Cochrane Database Syst. Rev..

[19] Slamon D.J., Leyland-Jones B., Shak S., Fuchs H., Paton V., Bajamonde A., Fleming T., Eiermann W., Wolter J., Pegram M. (2001). Use of chemotherapy plus a monoclonal antibody against HER2 for metastatic breast cancer that overexpresses HER2. N. Engl. J. Med..

[20] Seidman A., Hudis C., Pierri M.K., Shak S., Paton V., Ashby M., Murphy M., Stewart S.J., Keefe D. (2002). Cardiac dysfunction in the trastuzumab clinical trials experience. J. Clin. Oncol..

[21] Shanmuganathan J.W.D., Kragholm K., Tayal B., Polcwiartek C., Poulsen L.O., El-Galaly T.C., Fosbol E.L., D’Souza M., Gislason G., Kober L. (2021). Risk for Myocardial Infarction Following 5-Fluorouracil Treatment in Patients With Gastrointestinal Cancer: A Nationwide Registry-Based Study. JACC CardioOncol..

[22] Wacker A., Lersch C., Scherpinski U., Reindl L., Seyfarth M. (2003). High incidence of angina pectoris in patients treated with 5-fluorouracil. A planned surveillance study with 102 patients. Oncology.

[23] Jensen S.A., Hasbak P., Mortensen J., Sorensen J.B. (2010). Fluorouracil induces myocardial ischemia with increases of plasma brain natriuretic peptide and lactic acid but without dysfunction of left ventricle. J. Clin. Oncol..

[24] Akhtar S.S., Salim K.P., Bano Z.A. (1993). Symptomatic cardiotoxicity with high-dose 5-fluorouracil infusion: A prospective study. Oncology.

[25] Shyam Sunder S., Sharma U.C., Pokharel S. (2023). Adverse effects of tyrosine kinase inhibitors in cancer therapy: Pathophysiology, mechanisms and clinical management. Signal Transduct. Target. Ther..

[26] Ghatalia P., Morgan C.J., Je Y., Nguyen P.L., Trinh Q.D., Choueiri T.K., Sonpavde G. (2015). Congestive heart failure with vascular endothelial growth factor receptor tyrosine kinase inhibitors. Crit. Rev. Oncol. Hematol..

[27] Herrmann J., Yang E.H., Iliescu C.A., Cilingiroglu M., Charitakis K., Hakeem A., Toutouzas K., Leesar M.A., Grines C.L., Marmagkiolis K. (2016). Vascular Toxicities of Cancer Therapies: The Old and the New—An Evolving Avenue. Circulation.

[28] Choi H.D., Chang M.J. (2017). Cardiac toxicities of lapatinib in patients with breast cancer and other HER2-positive cancers: A meta-analysis. Breast Cancer Res. Treat..

[29] Dickerson T., Wiczer T., Waller A., Philippon J., Porter K., Haddad D., Guha A., Rogers K.A., Bhat S., Byrd J.C. (2019). Hypertension and incident cardiovascular events following ibrutinib initiation. Blood.

[30] Ghatalia P., Je Y., Kaymakcalan M.D., Sonpavde G., Choueiri T.K. (2015). QTc interval prolongation with vascular endothelial growth factor receptor tyrosine kinase inhibitors. Br. J. Cancer.

[31] Diaz-Serrano A., Gella P., Jimenez E., Zugazagoitia J., Paz-Ares Rodriguez L. (2018). Targeting EGFR in Lung Cancer: Current Standards and Developments. Drugs.

[32] Curigliano G., Lenihan D., Fradley M., Ganatra S., Barac A., Blaes A., Herrmann J., Porter C., Lyon A.R., Lancellotti P. (2020). Management of cardiac disease in cancer patients throughout oncological treatment: ESMO consensus recommendations. Ann. Oncol..

[33] Mahmood S.S., Fradley M.G., Cohen J.V., Nohria A., Reynolds K.L., Heinzerling L.M., Sullivan R.J., Damrongwatanasuk R., Chen C.L., Gupta D. (2018). Myocarditis in Patients Treated With Immune Checkpoint Inhibitors. J. Am. Coll. Cardiol..

[34] Cho J., Kim H.S., Ku B.M., Choi Y.L., Cristescu R., Han J., Sun J.M., Lee S.H., Ahn J.S., Park K. (2019). Pembrolizumab for Patients With Refractory or Relapsed Thymic Epithelial Tumor: An Open-Label Phase II Trial. J. Clin. Oncol..

[35] Salem J.E., Manouchehri A., Moey M., Lebrun-Vignes B., Bastarache L., Pariente A., Gobert A., Spano J.P., Balko J.M., Bonaca M.P. (2018). Cardiovascular toxicities associated with immune checkpoint inhibitors: An observational, retrospective, pharmacovigilance study. Lancet Oncol..

[36] Moslehi J.J., Salem J.E., Sosman J.A., Lebrun-Vignes B., Johnson D.B. (2018). Increased reporting of fatal immune checkpoint inhibitor-associated myocarditis. Lancet.

[37] Khunger A., Battel L., Wadhawan A., More A., Kapoor A., Agrawal N. (2020). New Insights into Mechanisms of Immune Checkpoint Inhibitor-Induced Cardiovascular Toxicity. Curr. Oncol. Rep..

[38] Pohl J., Mincu R.I., Mrotzek S.M., Hinrichs L., Michel L., Livingstone E., Zimmer L., Wakili R., Schadendorf D., Rassaf T. (2020). ECG Changes in Melanoma Patients Undergoing Cancer Therapy-Data From the ECoR Registry. J. Clin. Med..

[39] Abdul-Rahman T., Dunham A., Huang H., Bukhari S.M.A., Mehta A., Awuah W.A., Ede-Imafidon D., Cantu-Herrera E., Talukder S., Joshi A. (2023). Chemotherapy Induced Cardiotoxicity: A State of the Art Review on General Mechanisms, Prevention, Treatment and Recent Advances in Novel Therapeutics. Curr. Probl. Cardiol..

[40] Angsutararux P., Luanpitpong S., Issaragrisil S. (2015). Chemotherapy-Induced Cardiotoxicity: Overview of the Roles of Oxidative Stress. Oxid. Med. Cell. Longev..

[41] Ambrosio G., Zweier J.L., Duilio C., Kuppusamy P., Santoro G., Elia P.P., Tritto I., Cirillo P., Condorelli M., Chiariello M. (1993). Evidence that mitochondrial respiration is a source of potentially toxic oxygen free radicals in intact rabbit hearts subjected to ischemia and reflow. J. Biol. Chem..

[42] Fariss M.W., Chan C.B., Patel M., Van Houten B., Orrenius S. (2005). Role of mitochondria in toxic oxidative stress. Mol. Interv..

[43] Paradies G., Petrosillo G., Pistolese M., Di Venosa N., Federici A., Ruggiero F.M. (2004). Decrease in mitochondrial complex I activity in ischemic/reperfused rat heart: Involvement of reactive oxygen species and cardiolipin. Circ. Res..

[44] Paradies G., Petrosillo G., Pistolese M., Ruggiero F.M. (2000). The effect of reactive oxygen species generated from the mitochondrial electron transport chain on the cytochrome c oxidase activity and on the cardiolipin content in bovine heart submitochondrial particles. FEBS Lett..

[45] Lazzerini P.E., Capecchi P.L., Laghi-Pasini F. (2015). Long QT Syndrome: An Emerging Role for Inflammation and Immunity. Front. Cardiovasc. Med..

[46] Aromolaran A.S., Srivastava U., Ali A., Chahine M., Lazaro D., El-Sherif N., Capecchi P.L., Laghi-Pasini F., Lazzerini P.E., Boutjdir M. (2018). Interleukin-6 inhibition of hERG underlies risk for acquired long QT in cardiac and systemic inflammation. PLoS ONE.

[47] Ali A., Boutjdir M., Aromolaran A.S. (2018). Cardiolipotoxicity, Inflammation, and Arrhythmias: Role for Interleukin-6 Molecular Mechanisms. Front. Physiol..

[48] Li Q., Qin M., Tan Q., Li T., Gu Z., Huang P., Ren L. (2020). MicroRNA-129-1-3p protects cardiomyocytes from pirarubicin-induced apoptosis by down-regulating the GRIN2D-mediated Ca(2+) signalling pathway. J. Cell. Mol. Med..

[49] Sag C.M., Kohler A.C., Anderson M.E., Backs J., Maier L.S. (2011). CaMKII-dependent SR Ca leak contributes to doxorubicin-induced impaired Ca handling in isolated cardiac myocytes. J. Mol. Cell. Cardiol..

[50] Sutanto H., Lyon A., Lumens J., Schotten U., Dobrev D., Heijman J. (2020). Cardiomyocyte calcium handling in health and disease: Insights from in vitro and in silico studies. Prog. Biophys. Mol. Biol..

[51] Forstermann U., Munzel T. (2006). Endothelial nitric oxide synthase in vascular disease: From marvel to menace. Circulation.

[52] Taimeh Z., Loughran J., Birks E.J., Bolli R. (2013). Vascular endothelial growth factor in heart failure. Nat. Rev. Cardiol..

[53] Shi H., Gao Y., Dong Z., Yang J., Gao R., Li X., Zhang S., Ma L., Sun X., Wang Z. (2021). GSDMD-Mediated Cardiomyocyte Pyroptosis Promotes Myocardial I/R Injury. Circ. Res..

[54] Chintalgattu V., Ai D., Langley R.R., Zhang J., Bankson J.A., Shih T.L., Reddy A.K., Coombes K.R., Daher I.N., Pati S. (2010). Cardiomyocyte PDGFR-beta signaling is an essential component of the mouse cardiac response to load-induced stress. J. Clin. Investig..

[55] Hagiwara Y., Miyoshi S., Fukuda K., Nishiyama N., Ikegami Y., Tanimoto K., Murata M., Takahashi E., Shimoda K., Hirano T. (2007). SHP2-mediated signaling cascade through gp130 is essential for LIF-dependent I CaL, [Ca2+]i transient, and APD increase in cardiomyocytes. J. Mol. Cell. Cardiol..

[56] Villegas S., Villarreal F.J., Dillmann W.H. (2000). Leukemia Inhibitory Factor and Interleukin-6 downregulate sarcoplasmic reticulum Ca2+ ATPase (SERCA2) in cardiac myocytes. Basic Res. Cardiol..

[57] Tanaka T., Kanda T., Takahashi T., Saegusa S., Moriya J., Kurabayashi M. (2004). Interleukin-6-induced reciprocal expression of SERCA and natriuretic peptides mRNA in cultured rat ventricular myocytes. J. Int. Med. Res..

[58] Fontes J.A., Rose N.R., Cihakova D. (2015). The varying faces of IL-6: From cardiac protection to cardiac failure. Cytokine.

[59] Yamauchi-Takihara K., Kishimoto T. (2000). Cytokines and their receptors in cardiovascular diseases—Role of gp130 signalling pathway in cardiac myocyte growth and maintenance. Int. J. Exp. Pathol..

[60] Feldman A.M., Combes A., Wagner D., Kadakomi T., Kubota T., Li Y.Y., McTiernan C. (2000). The role of tumor necrosis factor in the pathophysiology of heart failure. J. Am. Coll. Cardiol..

[61] Cappetta D., Esposito G., Coppini R., Piegari E., Russo R., Ciuffreda L.P., Rivellino A., Santini L., Rafaniello C., Scavone C. (2017). Effects of ranolazine in a model of doxorubicin-induced left ventricle diastolic dysfunction. Br. J. Pharmacol..

[62] Ponde N.F., Lambertini M., de Azambuja E. (2016). Twenty years of anti-HER2 therapy-associated cardiotoxicity. ESMO Open.

[63] Xu T., Meng Q.H., Gilchrist S.C., Lin S.H., Lin R., Xu T., Milgrom S.A., Gandhi S.J., Wu H., Zhao Y. (2021). Assessment of Prognostic Value of High-Sensitivity Cardiac Troponin T for Early Prediction of Chemoradiation Therapy-Induced Cardiotoxicity in Patients with Non-Small Cell Lung Cancer: A Secondary Analysis of a Prospective Randomized Trial. Int. J. Radiat. Oncol. Biol. Phys..

[64] Finke D., Romann S.W., Heckmann M.B., Hund H., Bougatf N., Kantharajah A., Katus H.A., Muller O.J., Frey N., Giannitsis E. (2021). High-sensitivity cardiac troponin T determines all-cause mortality in cancer patients: A single-centre cohort study. ESC Heart Fail..

[65] Lv X., Pan C., Guo H., Chang J., Gao X., Wu X., Zhi X., Ren C., Chen Q., Jiang H. (2023). Early diagnostic value of high-sensitivity cardiac troponin T for cancer treatment-related cardiac dysfunction: A meta-analysis. ESC Heart Fail..

[66] Liu Y., Zhang T., Huang X., Shen L., Yang Q. (2024). Changes in Epicardial Adipose Tissue Assessed by Chest CT in Breast Cancer Patients Receiving Adjuvant Chemotherapy with Anthracyclines and Trastuzumab. Rev. Cardiovasc. Med..

[67] Rosenfeld R., Riondino S., Cerocchi M., Luciano A., Idone G., Lecis D., Illuminato F., Tolomei A., Torino F., Chiocchi M. (2024). Extracellular volume measured by whole body CT scans predicts chronic cardiotoxicity in breast cancer patients treated with neoadjuvant therapies based on anthracyclines: A retrospective study. Breast.

[68] Lustberg M.B., Reinbolt R., Addison D., Ruppert A.S., Moore S., Carothers S., Suresh A., Das H., Berger M., Ramaswamy B. (2019). Early Detection of Anthracycline-Induced Cardiotoxicity in Breast Cancer Survivors With T2 Cardiac Magnetic Resonance. Circ. Cardiovasc. Imaging.

[69] Thavendiranathan P., Shalmon T., Fan C.S., Houbois C., Amir E., Thevakumaran Y., Somerset E., Malowany J.M., Urzua-Fresno C., Yip P. (2023). Comprehensive Cardiovascular Magnetic Resonance Tissue Characterization and Cardiotoxicity in Women With Breast Cancer. JAMA Cardiol..

[70] Buck B., Chum A.P., Patel M., Carter R., Nawaz H., Yildiz V., Ruz P., Wiczer T., Rogers K.A., Awan F.T. (2023). Cardiovascular Magnetic Resonance Imaging in Patients With Ibrutinib-Associated Cardiotoxicity. JAMA Oncol..

[71] van Woerden G., van Veldhuisen D.J., Manintveld O.C., van Empel V.P.M., Willems T.P., de Boer R.A., Rienstra M., Westenbrink B.D., Gorter T.M. (2022). Epicardial Adipose Tissue and Outcome in Heart Failure With Mid-Range and Preserved Ejection Fraction. Circ. Heart Fail..

[72] Houbois C.P., Nolan M., Somerset E., Shalmon T., Esmaeilzadeh M., Lamacie M.M., Amir E., Brezden-Masley C., Koch C.A., Thevakumaran Y. (2021). Serial Cardiovascular Magnetic Resonance Strain Measurements to Identify Cardiotoxicity in Breast Cancer: Comparison With Echocardiography. JACC Cardiovasc. Imaging.

[73] Cardinale D., Ciceri F., Latini R., Franzosi M.G., Sandri M.T., Civelli M., Cucchi G., Menatti E., Mangiavacchi M., Cavina R. (2018). Anthracycline-induced cardiotoxicity: A multicenter randomised trial comparing two strategies for guiding prevention with enalapril: The International CardioOncology Society-one trial. Eur. J. Cancer.

[74] Avila M.S., Ayub-Ferreira S.M., de Barros Wanderley M.R., das Dores Cruz F., Goncalves Brandao S.M., Rigaud V.O.C., Higuchi-Dos-Santos M.H., Hajjar L.A., Kalil Filho R., Hoff P.M. (2018). Carvedilol for Prevention of Chemotherapy-Related Cardiotoxicity: The CECCY Trial. J. Am. Coll. Cardiol..

[75] Heck S.L., Mecinaj A., Ree A.H., Hoffmann P., Schulz-Menger J., Fagerland M.W., Gravdehaug B., Rosjo H., Steine K., Geisler J. (2021). Prevention of Cardiac Dysfunction During Adjuvant Breast Cancer Therapy (PRADA): Extended Follow-Up of a 2x2 Factorial, Randomized, Placebo-Controlled, Double-Blind Clinical Trial of Candesartan and Metoprolol. Circulation.

[76] Boekhout A.H., Gietema J.A., Milojkovic Kerklaan B., van Werkhoven E.D., Altena R., Honkoop A., Los M., Smit W.M., Nieboer P., Smorenburg C.H. (2016). Angiotensin II-Receptor Inhibition With Candesartan to Prevent Trastuzumab-Related Cardiotoxic Effects in Patients With Early Breast Cancer: A Randomized Clinical Trial. JAMA Oncol..

[77] Akpek M., Ozdogru I., Sahin O., Inanc M., Dogan A., Yazici C., Berk V., Karaca H., Kalay N., Oguzhan A. (2015). Protective effects of spironolactone against anthracycline-induced cardiomyopathy. Eur. J. Heart Fail..

[78] Gongora C.A., Drobni Z.D., Quinaglia Araujo Costa Silva T., Zafar A., Gong J., Zlotoff D.A., Gilman H.K., Hartmann S.E., Sama S., Nikolaidou S. (2022). Sodium-Glucose Co-Transporter-2 Inhibitors and Cardiac Outcomes Among Patients Treated With Anthracyclines. JACC Heart Fail..

[79] Avula V., Sharma G., Kosiborod M.N., Vaduganathan M., Neilan T.G., Lopez T., Dent S., Baldassarre L., Scherrer-Crosbie M., Barac A. (2024). SGLT2 Inhibitor Use and Risk of Clinical Events in Patients With Cancer Therapy-Related Cardiac Dysfunction. JACC Heart Fail..

